# An integrated *in vitro* carcinogenicity test that distinguishes between genotoxic carcinogens, non-genotoxic carcinogens, and non-carcinogens

**DOI:** 10.1093/mutage/geae004

**Published:** 2024-03-12

**Authors:** Katherine E Chapman, Ume-Kulsoom Shah, Jessica F Fletcher, George E Johnson, Shareen H Doak, Gareth J S Jenkins

**Affiliations:** Institute of Life Science, Swansea University Medical School, Swansea, SA2 8PP, United Kingdom; Institute of Life Science, Swansea University Medical School, Swansea, SA2 8PP, United Kingdom; Institute of Life Science, Swansea University Medical School, Swansea, SA2 8PP, United Kingdom; Institute of Life Science, Swansea University Medical School, Swansea, SA2 8PP, United Kingdom; Institute of Life Science, Swansea University Medical School, Swansea, SA2 8PP, United Kingdom; Institute of Life Science, Swansea University Medical School, Swansea, SA2 8PP, United Kingdom

**Keywords:** carcinogenicity, genotoxicity, micronucleus assay, multiple-endpoint, *in vitro*

## Abstract

Chemical safety testing plays a crucial role in product and pharmacological development, as well as chemoprevention; however, *in vitro* genotoxicity safety tests do not always accurately predict the chemicals that will be *in vivo* carcinogens. If chemicals test positive *in vitro* for genotoxicity but negative *in vivo*, this can contribute to unnecessary testing in animals used to confirm erroneous *in vitro* positive results. Current *in vitro* tests typically evaluate only genotoxicity endpoints, which limits their potential to detect non-genotoxic carcinogens. The frequency of misleading *in vitro* positive results can be high, leading to a requirement for more informative *in vitro* tests. It is now recognized that multiple-endpoint genotoxicity testing may aid more accurate detection of carcinogens and non-carcinogens. The objective of this review was to evaluate the utility of our novel, multiple-endpoint *in vitro* test, which uses multiple cancer-relevant endpoints to predict carcinogenic potential. The tool assessed micronucleus frequency, p53 expression, p21 expression, mitochondrial respiration, cell cycle abnormalities and, uniquely, cell morphology changes in human lymphoblastoid cell lines, TK6 and MCL-5. The endpoints were used to observe cellular responses to 18 chemicals within the following categories: genotoxic carcinogens, non-genotoxic carcinogens, toxic non-carcinogens, and misleading *in vitro* positive and negative agents. The number of endpoints significantly altered for each chemical was considered, alongside the holistic Integrated Signature of Carcinogenicity score, derived from the sum of fold changes for all endpoints. Following the calculation of an overall score from these measures, carcinogens exhibited greater potency than non-carcinogens. Genotoxic carcinogens were generally more potent than non-genotoxic carcinogens. This novel approach therefore demonstrated potential for correctly predicting whether chemicals with unknown mechanism may be considered carcinogens. Overall, while further validation is recommended, the test demonstrates potential for the identification of carcinogenic compounds. Adoption of the approach could enable reduced animal use in carcinogenicity testing.

## Introduction

The current battery of *in vitro* genotoxicity tests often does not reliably differentiate between chemical agents with carcinogenic potential and non-carcinogens (NCs). Misleading *in vitro* positive genotoxicity outcomes may result from *in vitro* test system oversensitivity, leading to chemicals testing positive in *in vitro* test systems but being negative when subsequently tested in *in vivo* models. Data continue to demonstrate that rates of misleading *in vitro* positive outcomes remain high [1]. Unnecessary animal testing can result from such misleading positive outcomes, leading to substantial ethical and financial implications [2]. It is therefore important that animal-based testing is reduced or replaced as far as is practical.

High rates of misleading *in vitro* positive outcomes may owe to several factors, including choice of cell type and dosing regimen [3–5]. Current tests typically only consider a limited number of relatively high doses of test agent and measure only genotoxicity endpoints. However, testing of multiple cellular endpoints for the same chemical can provide a more accurate overview of its genotoxic and carcinogenic potential and assist with the avoidance of misleading results [1, 6]. Holistic, integrated testing can therefore provide more robust datasets that represent the direct effects of chemicals, particularly if inter-supporting endpoints are assessed. This holistic approach can give a far wider picture of carcinogenic mechanisms, can show direct links between endpoints, and give more confidence in the final verdict of labelling a test agent a potential carcinogen. Furthermore, *in vitro* approaches in human cells may in some ways be superior to *in vivo* approaches, offering greater access to mechanistic analysis (e.g. gene expression endpoints, molecular initiating events [MIEs]), and larger quantities of test samples thanks to the use of immortalized cell lines [7].

While genotoxicity tests have been used extensively to assess the safety of chemicals, there is currently no *in vitro* test available for the specific detection of carcinogens that induce neoplastic events via non-genotoxic mechanisms. Non-genotoxic carcinogens (NGCs) constitute around 12% of all carcinogens [8], and there is a risk of assuming that a negative result in a micronucleus (MN) test *in vitro*, for example, can be equated to lack of carcinogenic potential, particularly as tests are also typically short-term (e.g. 4 or 24 h cellular exposure). It is therefore important that *in vitro* tests can identify NGCs as well as GCs.

High-throughput/high content (HT/HC) approaches are being recognized as being considerably more informative in terms of (geno)toxicity detection, with several new assays emerging (e.g. Litron’s Microflow, Toxys’ Toxtracker and Gentronix’s GreenScreen HC and BlueScreen HC). To this end, automation of the detection of genotoxicity is being recognized as beneficial for generating large datasets in reduced time, leading to greater statistical power [9–11]. Multiple endpoint/multiplexed approaches are also being recognized as being more informative methods for risk assessment, beyond the basic detection of genotoxicity as a single endpoint; such integrated approaches are already used *in vivo* [2] and indeed in industry, including AstraZeneca’s novel screening approaches [12]. These next-generation approaches are assisting with advancing the accurate detection of carcinogens, while preventing misleading *in vitro* positive outcomes.

## A novel, integrated *in vitro* tool for the prediction of carcinogenesis

**Figure 1.**
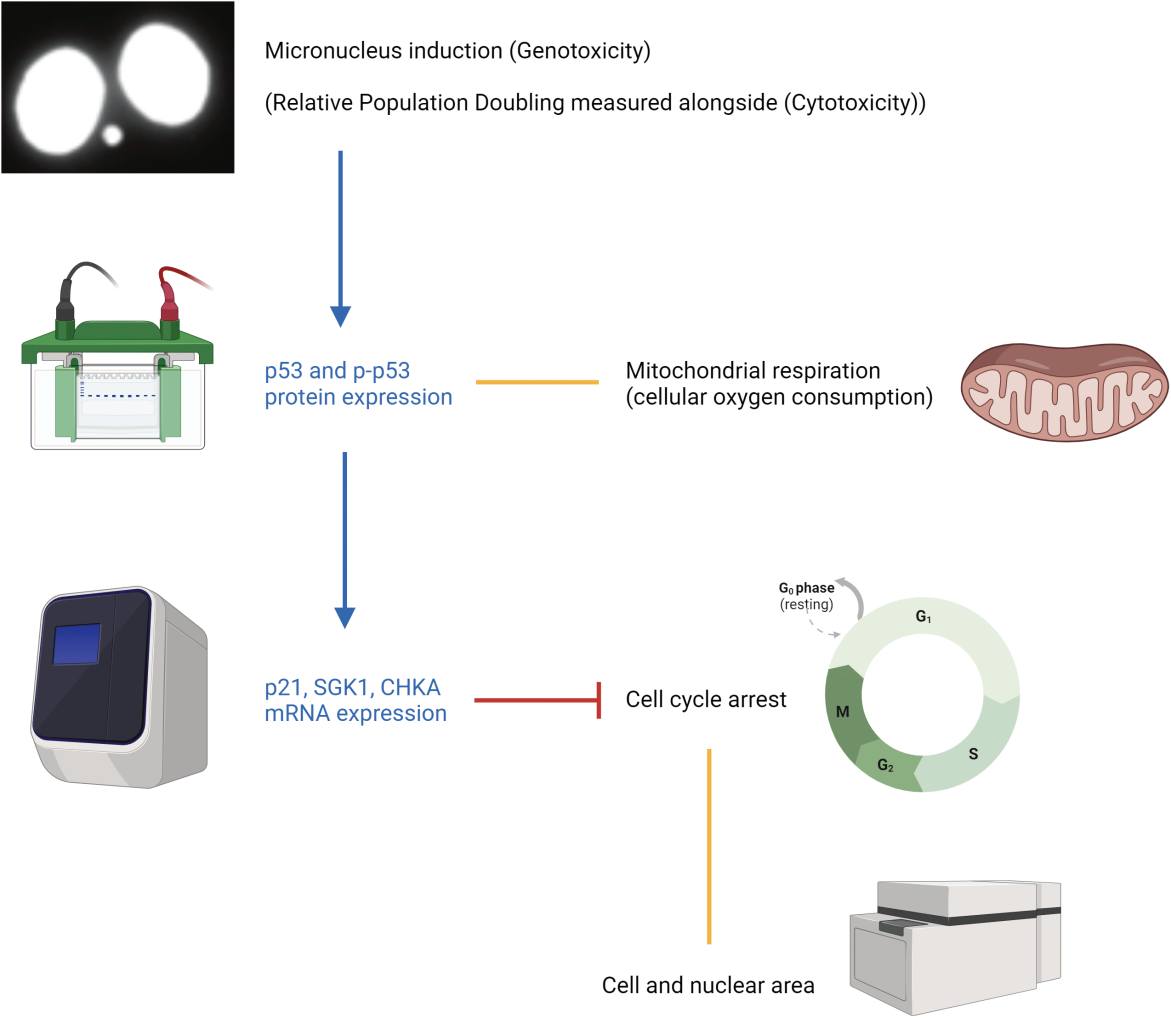
Summary of the molecular (blue text) and cellular (black text) endpoints used for the *in vitro* multiple-endpoint assay, and relationships between these endpoints. Arrows represent the series of events leading to gene upregulation (i.e. micronucleus induction leads to p53 accumulation, resulting in transcription of p21). The red line indicates cell cycle inhibition by p21, as part of the gene expression analysis. Orange lines represent a link involving at least one phenotypic endpoint; for example, p53 influences mitochondrial activity/apoptosis, and cell cycle arrest within a cell population may influence average cell and nuclear area values. Figure created in Biorender.

This review reports the findings of a long-term research project funded by a National Centre for the 3Rs (NC3Rs) Strategic Award in 2012. The project was established to develop a novel *in vitro* carcinogenicity tool involving the application of a range of cell biology endpoints to generate cancer-relevant data for multiple endpoints (Fig. 1). Six main endpoints were selected to encompass both molecular and cellular markers of the DNA damage response and early cellular changes associated with carcinogenesis. It was intended that such an approach would combine ‘traditional’ genotoxicity dose–response data with mechanistic data, such as cell signalling changes, as well as phenotypic changes associated with carcinogenesis. Endpoints were informed by the Hallmarks of Cancer, as identified by Hanahan and Weinberg [13, 14].

Since 2018, four primary research articles (Table 1) from the project have been published in peer-reviewed journals, documenting the results of the multiple endpoint study. In total, 18 chemical agents were tested in detail to investigate the assay’s potential for the detection of carcinogens and non-carcinogens. The first publication by Wilde *et al*. (2018) [15] compared the effects of genotoxic carcinogens (GCs) and non-genotoxic carcinogens (NGCs) on human cell lines. The publication by Shah *et al*. (2020) [16] centred on an *in vitro* negative chemical, whereas the publication by Chapman *et al*. (2021) [17] assessed additional carcinogens, non-carcinogens, and misleading *in vitro* positive chemicals. Data for a further genotoxic agent, CdCl_2_, was published by Stannard *et al*. (2023) [18].

**Table 1.** Summary of published articles documenting the results of the assessment of the novel *in vitro* carcinogenicity tool, including the chemicals tested and the overall results.

Citation	Chemicals studied	Summary of results
Wilde *et al*., 2018	**GCs:** Acetaldehyde Hydrogen peroxide (H_2_O_2_) Methyl methanesulfonate (MMS) Methyl nitrosourea (MNU) **NGCs:** Nickel chloride (NiCl_2_) Diethylhexyl phthalate (DEHP) Methyl carbamate 2,3,7,8-Tetrachlorodibenzodioxin (TCDD)	GCs altered a greater number of endpoints than NGCs. Both GCs and NGCs induced significant changes in at least two endpoints.
Shah *et al*., 2020	**Misleading *in vitro* negative:** Urethane	Urethane produced negative results for all endpoints. Testing in 3D liver models was positive.
Chapman *et al*., 2021	**GC:** Ochratoxin A **NGC:** β-oestradiol **Misleading *in vitro* positives:** 2,4-dichorophenol (2,4-DCP) Quercetin Quinacrine dihydrochloride (QDH) **Toxic non-carcinogens:** Caffeine Cycloheximide Phenformin HCl	Misleading *in vitro* positive and toxic non-carcinogens induced fewer significant changes of lower magnitude, when compared with carcinogens.
Stannard *et al*., 2023	**GC:** Cadmium chloride (CdCl_2_)	CdCl_2_ produced significant changes for multiple endpoints.

Chemicals were selected to include a broad range of different cellular mechanisms and chemical properties. Chemicals were also chosen with well-characterized *in vitro* and *in vivo* mechanisms (e.g. GCs, NGCs, etc., as outlined above), as well as agents with less well-defined mechanisms. Full methodological detail can be found within the publications included in Table 1.

For most test chemicals, the OECD-approved [19] human lymphoblastoid cell line TK6 was used. Where further metabolic capacity was required, in the case of 2,3,7,8-Tetrachlorodibenzodioxin (TCDD) and urethane, the MCL-5 cell line was also used. The use of human cells allows a species-specific analysis; TK6 are also p53-proficient, meaning greater genetic stability. The assays used were therefore short-term, with potential for some results (e.g. gene expression) being available after only 26–28 h (including cell harvesting and analysis). Low concentrations of test chemicals were used, with a maximum of 50% reduction in relative population doubling (RPD), relative to the control (100%), tolerated. The 50% reduction in RPD aligns with OECD guideline recommendations for the MN assay [19]. Genetic toxicity in the form of MN was used as a basis for dose selection for other endpoints, as MN is one of the current standards for genetic toxicity. This endpoint therefore also aligns with other endpoints, such as p53, due to the connection of both with the DNA damage response. As NGCs almost universally elicited negative MN responses, cytotoxicity was used in place of MN as an indicator of test chemical effects. It is acknowledged that basing dose selection on different endpoints for different chemical subtypes could lead to variability of results between these classes. However, it is noted that the lowest observed genotoxic effect level for MN results was generally approaching 50% RPD, helping to maintain some level of consistency.

Concentrations leading to a greater than 50% RPD reduction were excluded to avoid excessive toxicity confounding endpoint changes. Methods were kept consistent between the different publications to ensure that results for different chemicals were directly comparable. The micronucleus assay is the most widely used *in vivo* genotoxicity test [20], and therefore the micronucleus test was included in the new tool as the genotoxicity endpoint. The micronucleus data generated allowed the anchoring of doses of subsequent endpoints for the GCs. For NGCs, RPD was instead used to determine dose selection for further endpoints, in the absence of a statistically significant micronucleus response.

For all chemicals, cellular exposures were initially explored at 4 h; if there was a negative response (i.e. no reduction in cytotoxicity relative to the control) observed at 4 h, a 23-h timepoint was used for the same chemical for multiple-endpoint testing. This was to ensure that the effects of chemicals that required more time to induce cellular changes were not overlooked. Most chemicals’ effects were measured following 23 h exposures; five chemicals were studied at 4 h (three carcinogens, H_2_O_2_, CdCl_2_ and ochratoxin A, and two non-carcinogens, quercetin and cycloheximide) due to their ability to produce ‘early’ effects. As outlined previously, the analysis of multiple endpoints relating to carcinogenesis was predicted to lead to larger and more informative datasets for classifying chemical agents than current tests allow.

The objective of this review article is therefore to analyse the total data collected to date using the novel, multiple-endpoint approach, and evaluate the tool’s potential for correctly classifying chemicals based on their carcinogenic potential. It was hypothesized that the assay would correctly identify GCs on the basis that DNA-reactive mechanisms are directly associated with the chosen endpoints. Due to the design of the assay (i.e. multiple endpoints), it was predicted that the endpoints that were not exclusively associated with genotoxicity (e.g. cell cycle, cell morphology) may be altered by NGCs. It was predicted that NCs would alter few, if any, of the endpoints tested, helping to distinguish them from the carcinogens.

## GCs altered more endpoints than NGCs

Multiple endpoint testing was used to evaluate the potential of our novel approach for distinguishing carcinogens from non-carcinogens. As mentioned previously, a cohort of selected GCs and NGCs were tested for all endpoints, as well as a set of NCs for comparison, including misleading *in vitro* positives and toxic non-carcinogens. A misleading *in vitro* negative carcinogen, urethane, was included in the study; despite being a genotoxic carcinogen, urethane’s historical lack of geno(toxic) effects in *in vitro* tests led its data to be analysed separately.

**Figure 2.**
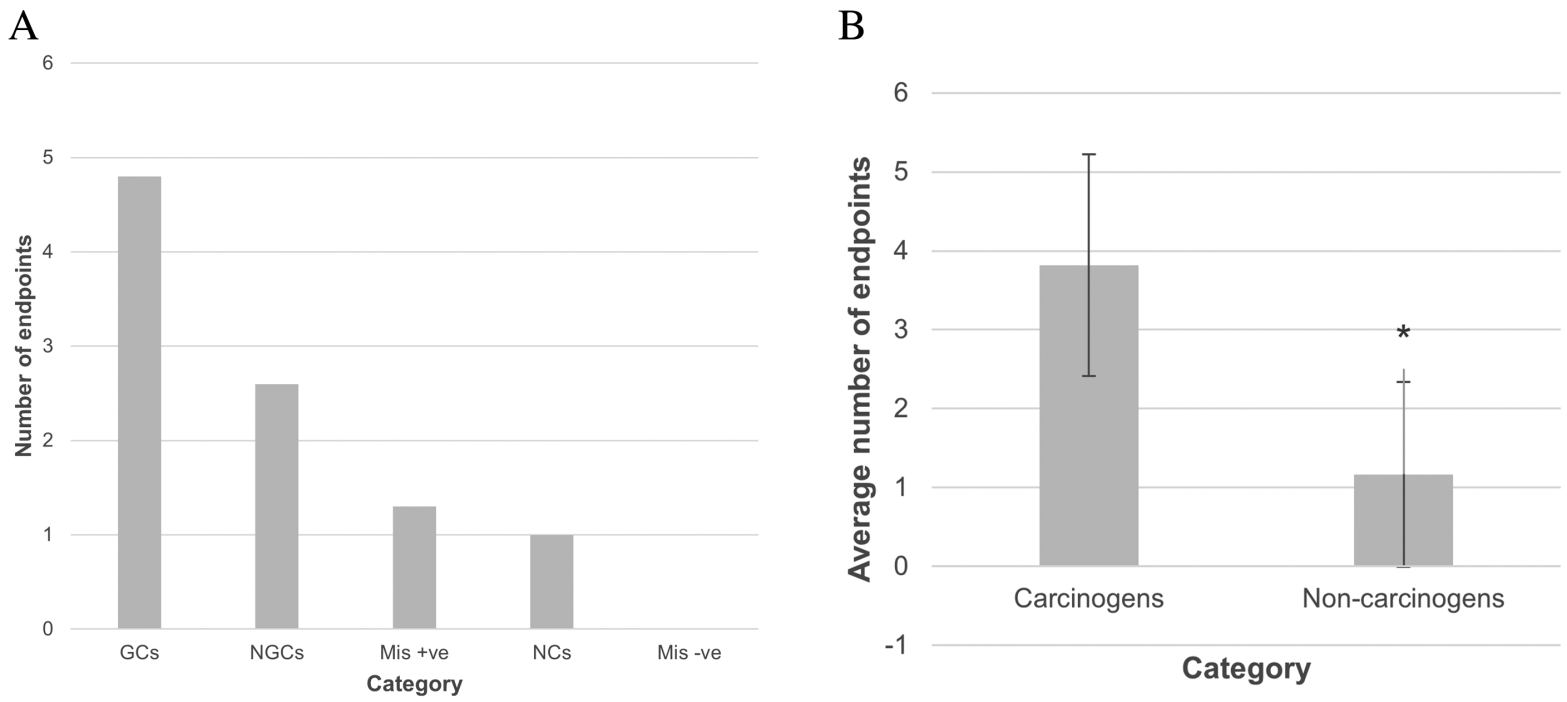
A. Average number of endpoints with statistically significant changes for each sub-category of test chemicals. GCs = Genotoxic carcinogens; NGCs = Non-genotoxic carcinogens; Mis + ve = misleading *in vitro* positives; NCs = Non-carcinogens (toxic); Mis -ve = Misleading *in vitro* negatives. B. Comparison of average endpoints significantly altered for all carcinogens (including urethane) and non-carcinogens. * - denotes *P* = .013.

Table 2 presents the overall results for chemicals that are classified as (*in vivo*) carcinogens. GC urethane tested negative for all endpoints (Table 3), and this will be discussed separately later in the review. In general, GCs demonstrated relatively high potency, inducing statistically significant changes for most endpoints tested (on average for the GC group, 4.8 out of 8 endpoints, Fig. 2). As the endpoints are generally interrelated and link directly to the mechanisms for many carcinogens, this outcome aligned with the overall study hypothesis. The relatively high number of endpoints altered by GCs supported the use of the chosen endpoints for identifying chemicals with genotoxic cellular effects. It is well established that alterations in p53, p21, and cell cycle dynamics (which influence cell and nuclear area) occur in response to genotoxic stress [21, 22], and even non-genotoxic stress [23].

**Table 2.** Summary of statistically significant changes to endpoints for genotoxic (GC) and non-genotoxic carcinogens (NGC) tested (denoted by + symbol). No change is represented by—symbol. The total number of positive outcomes is provided in the ‘Score’ column, and the average number of endpoints changed is indicated in the category average column. GC = Genotoxic carcinogens. NGC = Non-genotoxic carcinogens. ND = no data.

Type	Chemical	CBMN	p53	p21	CHKA	SGK1	Cell cycle	Cell morphology	Mito flux	Score
GC	MNU	+	+	+	-	+	+	+	-	6
GC	MMS	+	-	+	+	-	+	+	-	5
GC	H_2_O_2_	+	+	-	-	+	+	+	-	5
GC	Ochratoxin A	+	+	+	+	+	-	-	-	5
GC	Acetaldehyde	+	-	+	+	-	+	-	-	4
GC	CdCl_2_	+	+	-	-	-	+	+	ND	4
NGC	Methyl carbamate	-	-	+	+	+	-	+	-	4
NGC	NiCl_2_	-	+	-	-	-	+	+	-	3
NGC	DEHP	-	-	+	-	-	-	+	-	2
NGC	TCDD	-	-	-	+	+	-	-	-	2
NGC	Oestradiol	-	-	+	-	-	+	-	-	2

No change is represented by—symbol. The total number of positive outcomes is provided in the ‘Score’ column, and the average number of endpoints changed is indicated in the category average column. GC = Genotoxic carcinogens. NGC = Non-genotoxic carcinogens. ND = no data.

**Table 3.** Summary of statistically significant changes to endpoints for misleading *in vitro* positives (Mis + ve), non-carcinogens (NCs) and misleading *in vitro* negative (Mis -ve) chemicals tested (denoted by + symbol).

Type	Chemical	CBMN	p53	p21	CHKA	SGK1	Cell cycle	Cell morphology	Mito flux	Score
MP	Quercetin	+	+	-	-	-	+	-	-	3
NC	Caffeine	-	-	-	-	-	+	+	-	2
MP	2,4-DCP	-	-	-	-	-	-	-	+	1
NC	Cycloheximide	+	-	-	-	-	-	-	-	1
MP	QDH	-	-	-	-	-	-	-	-	0
NC	Phenformin HCl	-	-	-	-	-	-	-	-	0
MN	Urethane	-	-	-	-	-	-	-	-	0

No change is represented by—symbol. The total number of positive outcomes is provided in the ‘Score’ column, and the average number of endpoints changed is indicated in the category average column.

NGCs altered fewer endpoints than GCs overall, although all carcinogens (except urethane) significantly altered at least two of the endpoints. When the number of endpoints significantly altered by GCs (excluding urethane) was compared with NGCs, a two-tailed *t*-test produced a significant *P*-value of .001, indicating that the GCs altered significantly more endpoints than NGCs.

The NGCs’ diverse cellular mechanisms and lack of ability to induce genotoxicity directly explain the altering of fewer endpoints by this class of carcinogens. For example, TCDD is thought to contribute to carcinogenesis in its capacity as a tumour promoter [24], a property that may not be directly detected by the endpoints in this study. Going forward, it may be pertinent to incorporate endpoints that represent NGC mechanisms of action, particularly when these are better understood. Diversity in NGCs’ mechanisms may mean that it is more challenging to select endpoints that will successfully capture all NGCs. However, recent data have demonstrated that some chemicals considered to be NGCs may induce genotoxicity under certain chronic exposure conditions (data not shown).

The CBMN assay successfully distinguished between GCs and NGCs (excluding urethane), with all GCs testing positive (i.e. inducing a statistically significant increase in micronuclei above background (negative control) frequencies, *P* ≤ .05) and all NGCs testing negative for micronucleus induction (i.e. *P* > .05) (Table 2). This suggests that the CBMN assay may be used alone to reliably distinguish between GCs and NGCs, or alongside other endpoints. However, the CBMN assay alone will not allow NGCs to be distinguished from NCs, since both will usually test negative for such an endpoint.

Under the test conditions, mRNA encoding p21 was the endpoint most frequently altered by carcinogens and was significantly altered by 7 out of 11 carcinogens; both GCs and NGCs alike altered p21 mRNA expression. This observation contrasts with that of a previous study in TK6 by [25], where it was recognized that p21 ranked highly as an endpoint for distinguishing between GCs and NGCs, rather than being a universal indicator of carcinogenicity. H_2_O_2_ and CdCl_2_ were the only GCs in this study to test negative for p21 changes, although these chemicals did induce p53 protein and cell cycle changes.

Cell cycle abnormalities and changes to cell morphology were also frequently altered endpoints, with both significantly altered by 7 out of 11 carcinogens. Collectively, the observations relating to p21, cell cycle, and cell morphology changes suggest that cell cycle-related effects may be an effective indicator of early carcinogenesis. However, it is noted that one chemical, TCDD, did not alter p21, cell cycle, or morphology endpoints; TCDD was tested in MCL-5 cells rather than TK6, which may lead to slightly different cell cycle dynamics, although this difference may owe to TCDD’s tumour promoter status.

Interestingly, no carcinogen significantly altered all 8 endpoints, which may relate to the time-dependent nature of certain measures such as gene expression; wider time windows may be needed to observe some effects. The mitochondrial respiration abnormality endpoint tested negative with all carcinogens, suggesting that further optimization may be required or that this endpoint may be less useful in this context.

The DCFDA (dichlorodyhydrofluorescin diacetate) assay was used to measure reactive oxygen species (ROS) in cells following exposure to some of the test chemicals (GCs CdCl_2_, H2O2, MMS and MNU; NGCs DEHP, methyl carbamate and NiCl_2_) [15, 18]. Despite the use of a range of post-exposure timepoints (T + 10 min, 15 min, 30 min, 1 h, 2 h, 4 h, 6 h, and 24 h), a significant increase in fluorescence was only observed for NiCl_2_. This may link to NiCl2’s status as a ROS-inducer [26]. When used as a positive assay control, H_2_O_2_ significantly increased fluorescence; however, a positive response was only observed with a very high dose of 50 mM, far exceeding the selected test doses for H_2_O_2_. The negative results for most chemicals may be due to the low sensitivity of the DCFDA assay for the small-scale changes associated with low-dose exposures, rather than the absence of oxidative stress in cells. Due to results for this endpoint not being available for all chemicals, it was not included in the scoring.

## Non-carcinogens altered fewer endpoints than carcinogens

Table 3 presents the results for groups of NCs across the test endpoints and includes toxic NCs and *in vitro* ‘misleading’ positive chemicals. NCs generally altered fewer endpoints than carcinogens, as hypothesized, although they did not always produce fully negative results for all endpoints. On average, misleading *in vitro* positives altered 1.3 endpoints and toxic NCs altered 1 endpoint (Fig. 2A); these values were below averages for NGCs (2.6 endpoints) and GCs (4.8 endpoints). When carcinogens were compared with NCs (excluding urethane) via a *t*-test, carcinogens were found to alter significantly more endpoints than NCs (*P *= .001) (Fig. 2B). This distinction between carcinogens and NCs supports the use of the chosen endpoints for distinguishing between chemicals that are carcinogenic and those that are unlikely to pose carcinogenic risk.

Quercetin altered the greatest number of endpoints within the NC class (3 endpoints), while caffeine altered 2 endpoints; these were a similar number of endpoints to some of the less potent carcinogens tested. Cycloheximide and 2,4-DCP altered one endpoint each. Of the small number of endpoints significantly altered by NCs, it was noted that there was typically little pattern to the alterations; for example, cycloheximide and 2,4-DCP only altered micronucleus induction and mitochondrial flux, respectively. Cycloheximide significantly increased micronucleus induction at all concentrations within the dose range tested [17], despite being classed as a toxic NC. Cycloheximide is thought to cause stalling of translation and may therefore indirectly induce DNA damage [27], suggesting that it confer have genotoxic potential. It is acknowledged that positive results for test endpoints due to cytotoxicity alone could potentially be eliminated in future by further restricting the RPD range permitted for study; for example, only RPD values above 70% could be permitted rather than above 50%.

The fact that endpoints were altered by NCs in isolation further supports these not being true carcinogenic effects and could instead be associated with cytotoxicity, as supported by steep reductions in RPD values for these agents. It is likely that such outcomes are erroneous or misleading positive results.

As outlined above, quercetin was the most potent NC tested, altering 3 endpoints: MN, p53, and cell cycle. However, significant changes were typically only observed at the highest concentration tested and may, therefore, be due to cytotoxicity, adding support for the use of multiple doses in testing. Historically, quercetin has often produced positive results for genotoxicity *in vitro* [28], yet results for *in vivo* carcinogenicity were frequently negative [29, 30]. A possible explanation for negative *in vivo* results may be limited absorption and bioavailability *in vivo*, where a large proportion of quercetin is metabolized in the wall of the digestive tract and in the liver [31]. Therefore, most cells will not be exposed to notable levels of quercetin, and, as a result, outcomes might be dependent on the cell type analysed. Quercetin genotoxicity and carcinogenicity have therefore not been conclusively demonstrated [32], and this chemical’s positive outcomes may relate to its complex biological interactions. Overall, it is interesting that NGCs were negative for micronucleus induction, yet toxins may cause cellular toxicity that leads to micronucleus induction. This emphasizes the connection between toxic mechanisms and indirectly caused genotoxicity.

Given the overlap between quercetin, caffeine, and the carcinogens in terms of the number of endpoints altered, ranking via number of endpoints changed alone is insufficient for differentiation between different chemical classes. This suggested that an alternative measure to complement the number of endpoints would be valuable, and this will be explored in the following section.

**Figure 3.**
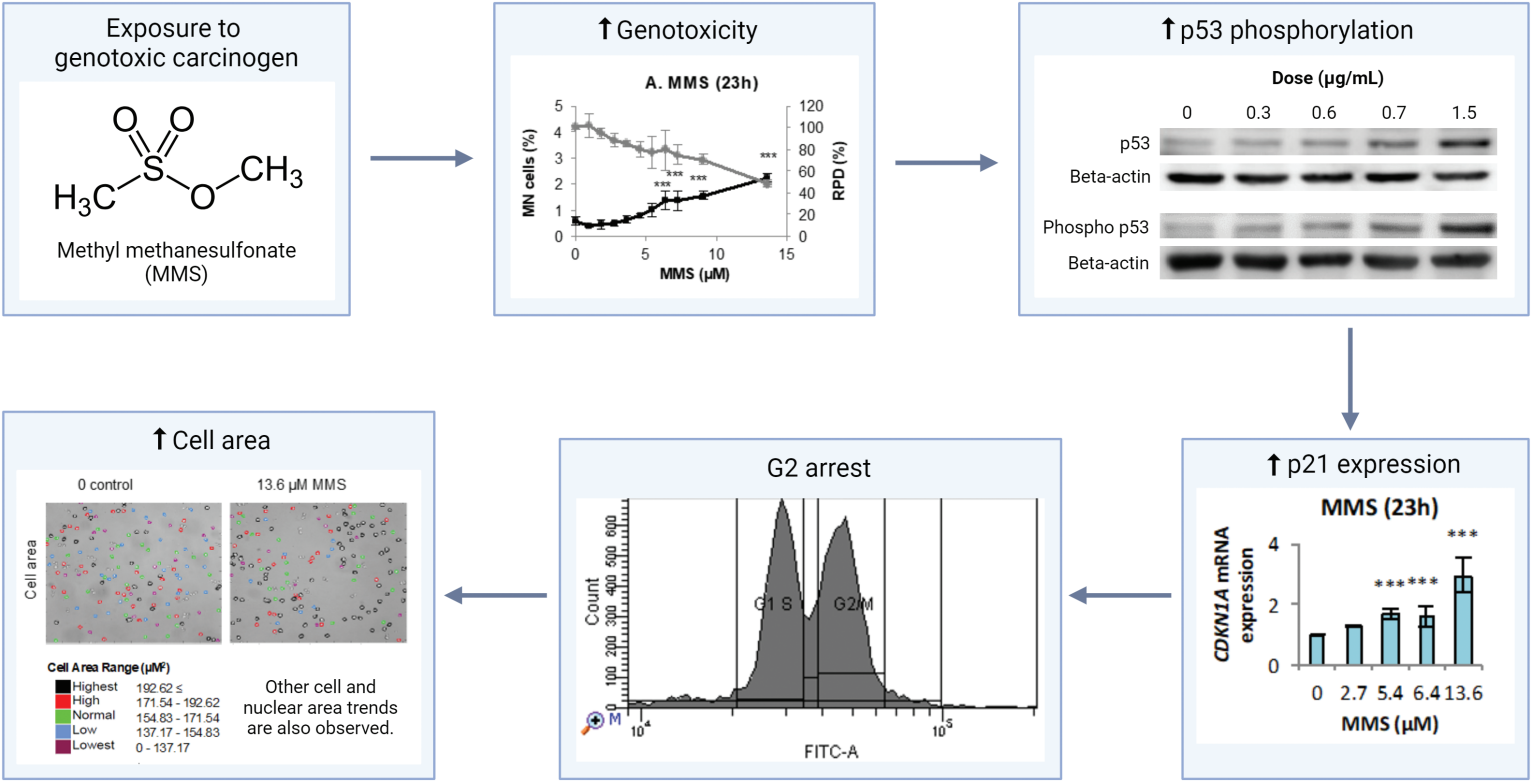
Flow diagram summarizing the relationship between the observed changes to test endpoints for MMS as an example GC. Figure created in Biorender.

Due to the interconnected nature of the tested endpoints, it may be possible to establish adverse outcome pathways (AOPs) for carcinogens; in particular, GCs appeared to consistently change certain endpoints, with some exceptions (Table 2). The affected endpoints and the direction of change (e.g. increase or decrease in cell area) were, in some cases, consistent between different chemicals; for example, alkylating agents MNU and MMS generally produced similar outcomes. Figure 3 illustrates the relationship between the respective endpoints for MMS as an example; an MIE for MMS could be DNA methylation, which leads to various key events (KEs). For example, the stabilization of p53 in response to DNA damage, leading to transcription of p21. As a cyclin-dependent kinase inhibitor (CDKN), p21 will then lead to cell cycle arrest at G2/M phases. Cell cycle arrest at the latter phases of the cell cycle will result in increased cell/nuclear area, hence cell morphology perturbations. Such an AOP model may therefore be useful for identifying MIEs that lead to larger-scale cellular changes (KEs/AOs), such as cell cycle and morphology perturbations. Consistent trends across these endpoints are likely to assist with the reliable detection of carcinogens, whereas inconsistencies or changes to isolated endpoints may indicate an erroneous or misleading positive result.

## Combining number of endpoints and integrated scores separated carcinogens from non-carcinogens

Generating numerical scores for chemical potency is a straightforward means of classifying chemicals for toxicity analysis and possibly stratifying test agents for follow-up investigation. Applications or programmes such as ToxPi GUI (Graphical User Interface) may be used to produce such scores and rank groups of chemicals following toxicity analysis [33]. Integrated signatures of carcinogenicity (ISCs) were scores generated to aid potency rankings for endpoints for our novel approach [15]. ISC values were calculated based on the sum of fold changes for all endpoints (including sub-endpoints) at the concentration inducing 50% RPD (Supplementary Table 1).

The ISC score provided an alternative approach to tallying the number of statistically significant changes in endpoints (Tables 2 and 3), as even non-significant variation in response relative to the negative control contributes to the overall score, which may be important for understanding the combined, or holistic, cellular effects of carcinogens. Furthermore, the use of *P* values as a threshold for biological effect may mean that effects close to significance are not considered.

**Figure 4.**
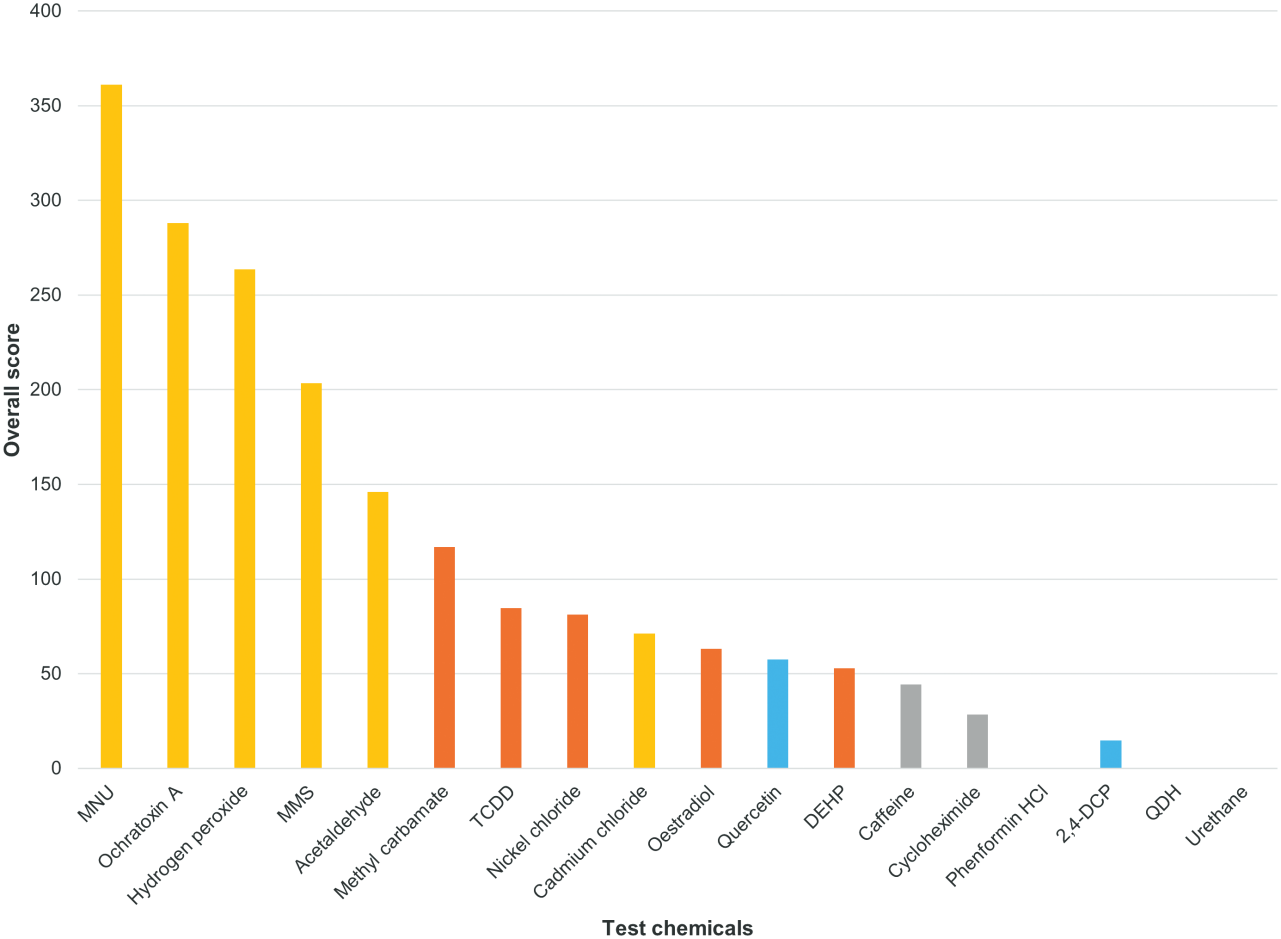
Summary of the overall scores for test chemicals based on ISC and number of endpoints changed. Chemicals are coloured according to sub-category; gold = GC, brown = NGC, orange = misleading *in vitro* negative; grey = toxic NC, blue = misleading *in vitro* positive. Chemicals with a 0 score and therefore not colour-coded were either misleading *in vitro* positives (Phenformin HCl, QDH) or negatives (urethane).

As observed in Supplementary Table 1, the number of endpoints changed, and ISCs were generated for each chemical. While the number of endpoints and the fold-change values are informative as individual scores, a combined overall score was generated by multiplying the score for the number of endpoints by the ISC value for each chemical (Fig. 4). This was intended to provide an overall impression of each chemical’s relative potency.

Ranking by ISC scores resulted in a general separation between carcinogens and NCs, with carcinogens generally producing higher scores than NCs. The ranking of overall scores, based on both number of endpoints and ISC, enabled almost complete separation of carcinogens from NCs, with carcinogens generally producing the highest scores. An exception was the misleading positive quercetin, which produced a slightly greater overall score than the lowest-scoring carcinogen, DEHP; however, there was only a 4.8 difference between these two chemicals. While quercetin did significantly alter 3 endpoints, its ISC score (19.2) remained lower than DEHP’s (26.4), suggesting that quercetin produced cellular changes of a lower magnitude. These results suggest that both number of endpoints changed and ISC should be considered when predicting carcinogenic potential; this also emphasizes the importance of testing multiple, rather than single, endpoints.

GCs generally produced greater overall scores than NGCs, with the five highest-ranking chemicals being GCs (excluding urethane). Overall scores for these ranged from 361.2 (MNU) to 146 (acetaldehyde). This suggested that the endpoints chosen were more sensitive for the detection of GCs than NGCs. An exception to this trend was the GC CdCl_2_, which produced an overall score of around 50% of acetaldehyde’s score. This lower potency may have partly owed to a lack of data for the mitochondrial endpoint for this chemical; however, assuming that this would have been one-fold as with other carcinogens, this would not substantially affect its overall score nor its ranking. CdCl_2_ did, however, produce a greater score than two of the NGCs, oestradiol and DEHP.

In terms of carcinogens, urethane was an anomaly and produced the lowest ISC score of all chemicals tested (12.7). Urethane also tested negative, or non-significant, for all endpoints in MCL-5 cells, despite these cells’ greater metabolic capacity when compared to cell line TK6 [16]. As discussed by Shah *et al*., it is possible that MCL-5 cells lacked the appropriate metabolic capacity for converting urethane to its genotoxic and carcinogenic form. Urethane did, however, induce genotoxicity in three-dimensional liver spheroid models, confirming that cell type and model may influence the capacity for urethane metabolism [16]. This makes a case for the use of multiple cell types/models for testing or following up negative results.

Overall, the results suggest that both a tally of significant endpoints affected and a holistic score based on fold changes should be considered in combination when classifying chemicals in terms of carcinogenic potential. Calculation of an overall score is also useful for ranking chemicals in terms of their potency. Our data suggest that a minimum of two endpoints should be altered as well as a minimum overall score, likely to be in the region of 50+, would be required for a chemical to be considered a possible carcinogen. However, a larger sample of chemicals would be required to define a threshold value for ISC; more data with a wider range of chemicals will better inform the tool and could allow ‘cut-off’ or threshold values for carcinogens to be established.

While others’ multiple-endpoint approaches have used genotoxicity, p53 and cell cycle endpoints, an endpoint unique to our tool is cell morphology. This agnostic marker of carcinogenicity is not thought to be linked to the Hallmarks of Cancer directly but is altered by many GCs and NGCs (7 of 11 carcinogens). Interestingly, of the NC and MP group, only one chemical, caffeine, significantly altered cell morphology. This suggests that cell morphology may be useful for the identification of carcinogens.

## Concluding remarks and future directions

There is a clear necessity for integrated approaches within standard *in vitro* genotoxicity testing protocols. Our new approach offers a rigorous, mechanism-centric alternative to simple *in vitro* genotoxicity and carcinogenicity testing of chemicals of unknown mechanism. Through testing of multiple endpoints for 18 chemicals, it was possible to successfully distinguish between carcinogens and NCs when the number of endpoints significantly altered, ISC scores, and an overall score, were considered. It was, however, noted that further NGC-specific endpoints may be beneficial as a follow-up for chemicals with borderline ISCs after the initial integrated test. Indeed, further cellular and molecular endpoints could be integrated to further develop the accurate identification of carcinogens. Nonetheless, it is acknowledged that carcinogenic potential is likely to be a spectrum, with all chemicals possessing some capacity for carcinogenesis. ISCs could potentially be aligned with other approaches such as benchmark dose models or modified use of ToxPi GUI. Recently published *in vitro* to *in vivo* dose extrapolation (IVIVE) methods could support cross-comparison of doses between different models and may be used to align with ISCs [34].

It was found that timepoints and cell types/models used may influence endpoint outcomes, leading to anomalous results, such as those associated with urethane. Further validation of the test with a greater number of chemicals, including nanomaterials and advanced materials, as well as additional timepoints and cell types/models would be beneficial to further validate the overall sensitivity and specificity of the test. Furthermore, advancements in imaging platforms, such as automated confocal microscopy, could assist with cell morphology analysis.

Changes observed could be used to construct relevant AOPs to inform risk assessment. Ultimately, it is projected that the application of this new approach will help to prevent unnecessary animal use within the carcinogenicity testing field.

## Supplementary Material

geae004_suppl_Supplementary_Tables_1

## Data Availability

No new data were generated in support of this publication.
