# Supplementary material for: An integrated in vitro carcinogenicity test that distinguishes between genotoxic carcinogens, non-genotoxic carcinogens, and non-carcinogens
Source: Mutagenesis. 2024 Mar 12;39(2):69–77. doi: 10.1093/mutage/geae004 (PMC10928837; doi:10.1093/mutage/geae004)
Supplement: geae004_suppl_Supplementary_Tables_1 [file geae004_suppl_supplementary_tables_1.docx]

**Supplementary Table 1.** Test chemical rankings according to the overall score, combining number of endpoints and ISC, for the concentration inducing 50% RPD. Chemicals are coloured according to sub-category; gold = GC, brown = NGC, orange = misleading *in vitro* negative; grey = toxic NC, blue = misleading *in vitro* positive. Adapted from Chapman *et al.* (2021).

| **Chemical** | **No. endpoints significantly changed** | **ISC score** | **Overall score** |
| --- | --- | --- | --- |
| MNU​ | 6 | 60.2 | 361.2 |
| Ochratoxin A​ | 5 | 57.6 | 288 |
| H_2_O_2_ | 5 | 52.7 | 263.5 |
| MMS​ | 5 | 40.7 | 203.5 |
| Acetaldehyde​ | 4 | 36.5 | 146 |
| Methyl carbamate​ | 4 | 29.2 | 116.8 |
| TCDD​ | 2 | 42.3 | 84.6 |
| NiCl_2_ | 3 | 27.1 | 81.3 |
| CdCl_2_ | 4 | 17.8 | 71.2 |
| Oestradiol​ | 2 | 31.6 | 63.2 |
| Quercetin​ | 3 | 19.2 | 57.6 |
| DEHP​ | 2 | 26.4 | 52.8 |
| Caffeine​ | 2 | 22.2 | 44.4 |
| Cycloheximide​ | 1 | 28.4 | 28.4 |
| Phenformin HCl​ | 0 | 16.2 | 0 |
| 2,4-DCP | 1 | 14.8 | 14.8 |
| QDH​ | 0 | 13 | 0 |
| Urethane​ | 0 | 12.7 | 0 |
